# Molecular Hydrogen Inhibits Colorectal Cancer Growth via the AKT/SCD1 Signaling Pathway

**DOI:** 10.1155/2022/8024452

**Published:** 2022-04-26

**Authors:** Xiangyan Zhang, Geru Tao, Ya'nan Zhao, Sijie Xing, Jie Jiang, Boyan Liu, Shucun Qin

**Affiliations:** ^1^Department of Pathophysiology, Basic Medicine College, Qingdao University, Qingdao 266000, China; ^2^The Second Affiliated Hospital of Shandong First Medical University, Tai'an 271000, China; ^3^Taishan Institute for Hydrogen Biomedicine, Shandong First Medical University and Shandong Academy of Medical Sciences, Tai'an 271000, China

## Abstract

**Objective:**

Molecular hydrogen (H_2_) has been considered a potential therapeutic target in many cancers. Therefore, we sought to assess the potential effect of H_2_ on colorectal cancer (CRC) in this study.

**Methods:**

The effect of H_2_ on the proliferation and apoptosis of RKO, SW480, and HCT116 CRC cell lines was assayed by CCK-8, colony formation, and flow cytometry assays. The effect of H_2_ on tumor growth was observed in xenograft implantation models (inhalation of 67% hydrogen two hours per day). Western blot and immunohistochemistry analyses were performed to examine the expression of p-PI3K, PI3K, AKT, pAKT, and SCD1 in CRC cell lines and xenograft mouse models. The expression of SCD1 in 491 formalin-fixed, paraffin-embedded CRC specimens was investigated with immunochemistry. The relationship between SCD1 status and clinicopathological characteristics and outcomes was determined.

**Results:**

Hydrogen treatment suppressed the proliferation of CRC cell lines independent of apoptosis, and the cell lines showed different responses to different doses of H_2_. Hydrogen also elicited a potent antitumor effect to reduce CRC tumor volume and weight *in vivo*. Western blot and IHC staining demonstrated that H_2_ inhibits CRC cell proliferation by decreasing pAKT/SCD1 levels, and the inhibition of cell proliferation induced by H_2_ was reversed by the AKT activator SC79. IHC showed that SCD1 expression was significantly higher in CRC tissues than in normal epithelial tissues (70.3% vs. 29.7%, *p* = 0.02) and was correlated with a more advanced TNM stage (III vs. I + II; 75.9% vs. 66.3%, *p* = 0.02), lymph node metastasis (with vs. without; 75.9% vs. 66.3%, *p* = 0.02), and patients without a family history of CRC (78.7% vs. 62.1%, *p* = 0.047).

**Conclusion:**

This study demonstrates that high concentrations of H_2_ exert an inhibitory effect on CRC by inhibiting the pAKT/SCD1 pathway. Further studies are warranted for clinical evaluation of H_2_ as SCD1 inhibitor to target CRC.

## 1. Introduction

Colorectal cancer (CRC) is one of the most common cancers, and its morbidity has increased steadily in recent years [1]. The current treatment strategy is main based on surgery, chemotherapy, and targeted treatment, and the outcome of CRC patients has significantly improved due to anticancer therapy [2]. However, some patients have a high incidence of adverse effects caused by surgery and chemotherapy [3]. Therefore, it is important to explore new therapeutic strategies for CRC patients.

Molecular hydrogen (H_2_), which exerts antioxidant, anti-inflammatory, and antiapoptotic properties, has been implemented to treat various diseases, including inflammation [4], ischemia/reperfusion injury [5], atherosclerosis [6], and tumors [7]. Hydrogen molecules can directly diffuse into the cytosol, mitochondria, and nucleus due to their permeability to cell membranes. Several intake methods, such as inhaling hydrogen, drinking hydrogen-rich water (HRW), and injecting hydrogen-saturated saline, are valid and reliable for treatment [7–9]. Reports have shown that H_2_ can inhibit tumor cell proliferation, invasion, and migration via various molecular pathways. Wang et al. demonstrated that 60% H_2_ could inhibit lung cancer migration and invasion by maintaining chromosome stability [10]. Yang et al. found that drinking HRW could inhibit endometrial cancer growth via ROS/NLRP3/caspase-1/GSDMD-mediated pyroptosis *in vivo* [11]. A study from Japan showed that inhalation of H_2_ for three h/day could improve the prognosis of advanced CRC patients by increasing the number of PD-L1/CD8^+^ cells [7]. Recently, Ma et al. argued that H_2_ could regulate glioma stem-like cell differentiation and inhibit glucose metabolism in glioblastoma multiforme (GBM) tumors [12], which provides a new perspective on H_2_ and tumor metabolism.

Metabolic pathways associated with oncogenesis and tumor metastasis have been explored in recent years, and targeting metabolism is a novel strategy for cancer treatment [13, 14]. Abnormal metabolic changes, including glucose uptake, glycolysis, and lactic acid production, are responsible for tumor growth, proliferation, and immune escape [13]. We previously observed that the HRW decreased serum oxidized low-density lipoprotein level and reduced ROS accumulation in the atherosclerosis model of *Ldlr−/−* mice [15]. Moreover, hydrogen reduced the levels of lipid peroxidation (LOP) and increased the activity of superoxide dismutase (SOD) and free fatty acids [16]. These results strongly suggested that H_2_ presents improved effects against lipoprotein metabolize. Stearoyl-CoA desaturase 1 (SCD1) is the rate-limiting enzyme of fatty acid biosynthesis (FAS) that catalyzes the conversion of saturated fatty acids (SFAs) into monounsaturated fatty acids (MUFAs), which are one of the critical components of triglycerides and membrane phospholipids. SCD1 have been found in many tumor tissues, including colorectal, gastric, breast, and lung cancer [17]. In addition, increased expression of SCD1 has been demonstrated to be correlated with cancer progression and poor prognosis in cancer patients [18, 19]. Moreover, SCD1 has been shown to be a marker of cancer stem cells (CSCs) in CRC [20]. An SCD1 inhibitor diminished the stemness of CSCs and reduced ovarian CSC proliferation *in vivo* [21]. Thus, SCD1 appears to be a promising anticancer target. We previously observed that H_2_ could reduce SCD1 expression in high-fat diet mediated live injury rat model. This result prompts us to speculate that hydrogen could inhibit the proliferation of colorectal cancer cells by reducing the expression of SCD1. So, we conducted a study to explore the potential role of H_2_ in the suppression of tumor proliferation of colorectal carcinoma cells.

Interestingly, we found that H_2_ could inhibit colorectal carcinoma cell proliferation by downregulating the AKT/SCD1 pathway. Our study also revealed that SCD1 is highly expressed in CRC patients in eastern China. Therefore, H_2_ may be a choice for CRC patients who harbor tumors with overexpression of SCD1.

## 2. Materials and Methods

### 2.1. Tissue Samples and Clinic Data

Four hundred ninety-one formalin-fixed, paraffin-embedded CRC specimens with matched adjacent normal epithelium tissues from CRC patients who underwent primary surgical resection from 2014 to 2016 in the Affiliated Hospital of Qingdao University were selected for this study. Patients who had undergone preoperative radiotherapy, chemotherapy, and/or targeted therapy were not included. The clinical and pathological variables were collected as previously described [22]. The patients were followed up until December 2020, and data concerning cancer recurrence and patient survival were collected. This study was approved by the Ethics Committee of the Shandong First Medical University and Shandong Academy of Medical Sciences (W202107060302) and the Affiliated Hospital of Qingdao University (QDFY-20130049).

### 2.2. Cell Lines and Cell Cultures

The human CRC cell lines RKO, SW480, and HCT116 were obtained from the Cell Bank of the Chinese Academy of Sciences (Shanghai, China) and were cultured in Dulbecco's modified Eagle's medium (DMEM; Gibco, C11995500BT, Thermo Fisher, USA) supplemented with 10% -30% fetal bovine serum (FBS; Gibco, 10099-141c, Thermo Fisher, USA), 100 IU penicillin, and 100 mg/mL streptomycin. All cells were incubated in a humidified atmosphere at 37°C with 5% CO2. For H_2_ treatment (hydrogen machine; Wuxi Puhe, pH-1-A1 China), cells were cultured in 30% (30% H_2_, 5% CO_2_, 21% O_2_, and 44% N_2_), 50% (50% H_2_, 5% CO_2_, 21% O_2_, and 24% N_2_), or 70% H_2_ (70% H_2_, 5% CO_2_, 21% O_2_, and 4% N_2_), with 5% CO_2_ (5% CO_2_, 21% O_2_, and 74% N_2_) as the control conditions (Ctrl group). The AKT activator SC79 (HY-18749) was purchased from MedChemExpress (China).

### 2.3. Western Blot Analysis

Cells were lysed in RIPA buffer (CW2333S) supplemented with protease inhibitor cocktail (CW2200S) and phosphatase inhibitors (CW2383S). Total protein was extracted from tissue samples by a Tissue Protein Extraction Kit (CW0891M) (all from CWBIO, Beijing, China). Then, the protein concentration was determined using a BCA protein assay kit (Beyotime). Total proteins from cells (20 *μ*g) and tissues (20 *μ*g) were separated by SDS PAGE through a 10% gel and transferred to a polyvinylidene fluoride (PVDF) membrane (Millipore, IPVH00010, MA, USA) at 100 V for 60 min. After blocked with Tris-buffered saline containing Tween-20 (TBST, 1000 : 1) and 5% fat-free milk for 2 h, the membranes were incubated at 4°C overnight with primary antibodies against SCD1 (1 : 1000; Bioss, Cat# bs-3787R, Beijing, China), p-PI3K (1 : 1000; Cat#: 17366; Cell Signaling Technology, Danvers, MA, USA), PI3K (1 : 1000; clone: 1F6A7; Proteintech, Wuhan, China), phospho-AKT (Ser473; 1 : 1000; Cat# 4069; Cell Signaling Technology, Danvers, MA, USA), AKT (1 : 1000; Cat# 4691; Cell Signaling Technology), and actin (rabbit polyclonal; 1 : 2000; Cat# E-AB-20058; Elabscience, Wuhan, China).

### 2.4. Cell Proliferation Assay

Cell proliferation was analyzed with a Cell Counting Kit-8 assay (Cat. CK04, Dojindo, Japan). Briefly, cells were seeded in 96-well plates at a density of 1 × 10^3^ cells per well and cultured for 5 days. The absorbance at 450 nm was measured in real-time every 24 h after incubation with 10 *μ*L of CCK-8 reagent and 90 *μ*L of cell culture medium for 2 h at 37°C. Light absorbance was measured by a microplate reader (Infifinit f200, Tecan, Australia). Experiments were performed independently at least 3 times.

### 2.5. Colony Formation Assay

For the colony formation assay, RKO, SW480, and HCT116 cells were seeded in 6-well plates at 1 × 10^3^ cells per well and cultured for 14 days. The medium was replaced every 3 days. Cell colonies were washed twice with PBS, fixed with 4% paraformaldehyde for 30 min, and stained with 0.1% crystal violet for 1 min. The experiment was repeated at least 3 times.

### 2.6. Apoptosis Analysis

Cells were cultured in 6-well plates, washed twice with cold PBS, and treated with 0.25% trypsin digestion solution without EDTA (T1350, Solarbio, China). The cells were collected and resuspended in 500 *μ*L of binding buffer (Cat. No. KGA107, KeyGEN BioTechnology, China). Then, 5 *μ*L of Annexin V-FITC and 5 *μ*L of propidium iodide were added to the buffer for staining for 10 min at room temperature. Cells were stained with PI and analyzed by flow cytometry (BD FACS Calibur).

### 2.7. Animal Experiments

A murine xenograft model was developed to investigate the effects of H_2_ on tumor proliferation *in vivo*. Four-week-old BALB/c- nude mice were purchased from Charles River (Beijing, China) and injected subcutaneously with 4 × 10^6^ viable RKO cells. After injection, the mice were randomly assigned to 2 groups: the hydrogen inhalation group (66% H_2_ and 33% O_2_) and the control group (66% N_2_ and 33% O2) (hydrogen machine; Huimei, China). The mice in the inhalation group were exposed to H_2_ for 2 hours every day. Tumor growth was examined for three weeks. Tumor volume (*V*) was monitored by measuring the length (*L*) and width (*W*) of the tumor following the equation *V* = (*L* × *W*^2^) × 0.5. Protocols involving animal experiments were approved by Shandong First Medical University.

### 2.8. Immunohistochemistry

Paraffin-embedded tissues were deparaffinized and rehydrated with xylene and a graded ethanol series. Sections were treated with 3% hydrogen peroxidase (10 min, room temperature), and antigen retrieval was performed with EDTA (pH = 8.0) for 10 min; the sections were then incubated with primary antibodies targeting p-PI3K (1 : 600; Cat#: 17366; Cell Signaling Technology, Danvers, MA, USA), PI3K (1 : 600; clone: 1F6A7; Proteintech, Wuhan, China), SCD1 (1 : 400; Bioss, Cat# bs-3787R, Beijing, China), phospho-AKT (Ser473; 1 : 600; Cat# 4069; Cell Signaling Technology, Danvers, MA, USA), and AKT (1 : 400; Cat# 4691; Cell Signaling Technology) for 1 hour at 37°C followed by incubation with secondary antibody for 30 min at 37°C and rinses with PBS. Then, sections were developed with a 3,3-diaminobenzidine (DAB) kit (ZSGB-Bio, ZL1-9017, Beijing, China) and counterstained with hematoxylin.

All slides were examined and graded by two pathologists blinded to the clinical diagnosis. Cytoplasmic SCD1 was quantified based on the extent of positive tumor cells and the intensity the staining. The percentage of positively stained tumor cells was scored as 0 (negative), 1 (1 − 25%), 2 (26–50%), 3 (51–75%), and 4 (>75%). The two scores were multiplied, and the resulting immune-reactive score (IRS) (values from 0 to 12) was used to classify the samples into two categories: high (4–12) and low (1–3) [23].

### 2.9. Statistical Analysis

All data were evaluated using the SPSS 19.0.0 software (SPSS, Chicago, IL, USA) and expressed as the mean ± SD. All experiments were repeated at least 3 times. The relationship between the clinicopathological features and SCD1 expression, as well as the differences in SCD1 expression between normal epithelium and CRC tissues, was evaluated using a chi-square test or Fisher's exact test. Survival analysis for disease-free survival (DFS) and overall survival (OS) was estimated using Kaplan–Meier analysis with the log-rank test. Multivariable analysis was performed using Cox regression. A probability (*P*) value <0.05 was considered statistically significant.

## 3. Results

### 3.1. Hydrogen Inhibits CRC Cell Proliferation *In Vitro* in a Dose-Dependent Manner

**Figure 1:**
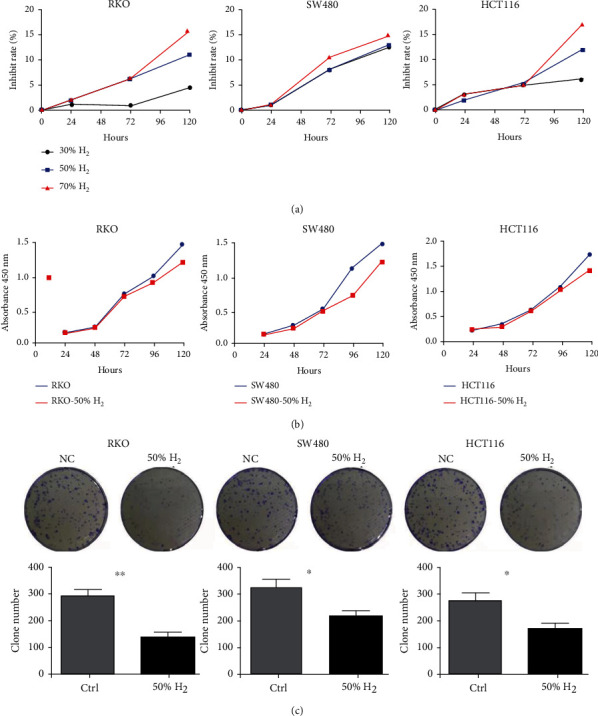
Hydrogen inhibits CRC cell proliferation in vitro. As shown in (a), the proliferation results demonstrated that H_2_ had a good inhibitory effect on SW480 cells, even at low concentrations (30%), while only high concentrations (more than 50%) of H_2_ suppressed RKO and HCT116 cells growth, as determined by the CCK-8 assay. (b) 50% hydrogen decreased cell viability compared to the ctrl group in a time-dependent manner at 24, 48, 72, 96, and 120 h measured spectrophotometrically at 450 nm by CCK8 assay in RKO, SW480, and HCT116 cells. (c) The colony formation assay showed that 50% H_2_ decreased the number of colonies arising from RKO, SW480, and HCT116 cells.

To explore the potential effect of H_2_ on CRC, we evaluated the effect of different concentrations of H_2_ on the growth of RKO, SW480, and HCT116 cells. As shown in Figure 1(a), the proliferation results demonstrated that H_2_ had a potent inhibitory effect on SW480 cells, even at low concentrations (30%), while only high concentrations (more than 50%) of H_2_ suppressed RKO and HCT116 cells growth, as indicated by the CCK-8 assay (Figure 1(a)). There, we adopted 50% H_2_ for subsequent experiments. The CCK8 and colony formation assay showed that 50% H_2_ decreased the colony number of CRC cells (Figures 1(b) and 1(c)). To determine whether the inhibitory effect of H_2_ on cell proliferation is associated with apoptosis, we performed flow cytometry to explore the apoptosis after treatment with H_2_. Time-course analysis indicated that H_2_ did not promote apoptosis in CRC cells (Supplement Materials). These results demonstrate that H_2_ inhibits the proliferation of CRC cells *in vitro* in a dose-dependent manner.

### 3.2. Hydrogen Acts as a Tumor Suppressor of CRC *In Vivo*

**Figure 2:**
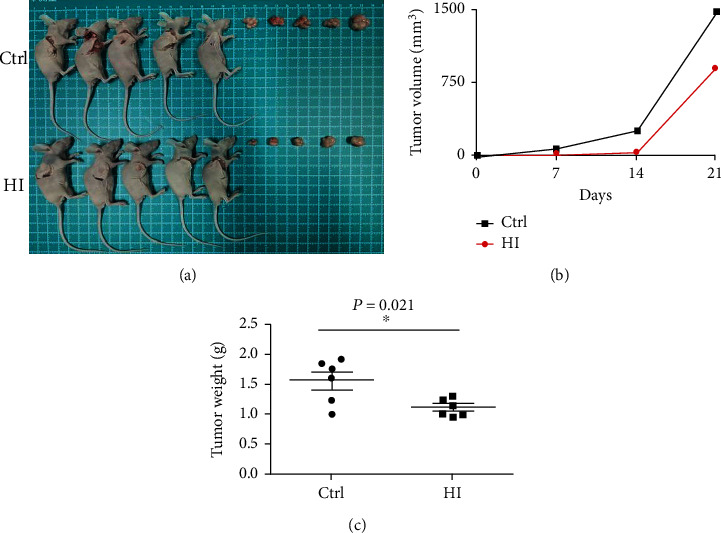
Hydrogen acts as a tumor suppressor of CRC in vivo. As shown in (a–c), tumor growth was significantly suppressed in nude mice administered inhalational H_2_. Hydrogen inhalation significantly reduced the tumor weight (1.11 ± 0.06 g vs. 1.56 ± 0.15 g, *p* = 0.021) and volume (898 ± 154 mm^3^ vs. 1413 ± 138 mm^3^, *p* = 0.032) compared to those in control mice.

Next, we evaluated the effect of H_2_ on CRC cell growth in a xenograft model. A total of 10 mice were injected with RKO cells. A transparent closed box (40 cm × 20 cm × 20 cm) connected to a hydrogen-oxygen nebulizer machine (Asclepius Meditec Inc., Shanghai, China) that produces an atmosphere containing 67% H_2_ and 33% O_2_ (*V*/*V*) was applied. Hydrogen treatment was administered from the second day after injection to the end of the experiment. Animals were placed in this box and inhaled the mixture for 2 h per day. During this time, the mice were awake and freely moving. A TRACE GC Ultra Gas Chromatograph (Thermo Fisher, MA, USA) was used to monitor the concentration of hydrogen gas in the closed box. The control group was exposed to mixed air, which was composed of 67% N_2_ and 33% O_2_. According to our observations, the tumors were visible starting on day 7 after injection. The tumors exhibited irregularly exophytic, circumscribed, and cauliflower-like surface from day 7 onward. Mice were sacrificed, and the tumors were excised on day 21. As shown in Figure 2, tumor growth was significantly suppressed in nude mice that inhaled H_2_. Hydrogen inhalation significantly reduced the tumor weight (1.11 ± 0.06 g vs. 1.56 ± 0.15 g, *p* = 0.021) and volume (898 ± 154 mm^3^ vs. 1413 ± 138 mm^3^, *p* = 0.032) compared to those in control mice.

### 3.3. Hydrogen Inhibits CRC Cell Proliferation by Decreasing pAKT/SCD1

**Figure 3:**
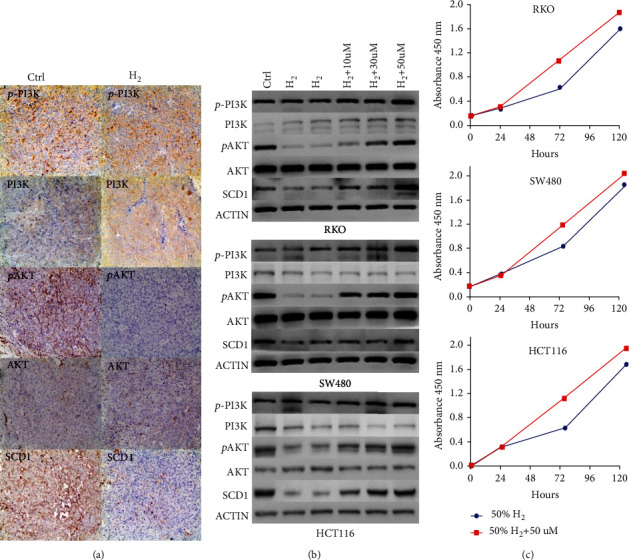
Hydrogen inhibits CRC cell proliferation by decreasing pAKT/SCD1. (a) IHC assay in tumors (RKO cells) after H_2_ treatment and control (100x). (b) Western blot analysis showing the expression levels of p-PI3K, PI3K, AKT, pAKT, and SCD1 in RKO, SW480, and HCT116 cells before and after H_2_ treatment. Western blotting assays showed that the expression of SCD1 was significantly upregulated and presented a dose-dependent effect with SC79 treatment. (c) The inhibition of cell proliferation induced by H_2_ was reversed when cells were treated with SC79, as determined by the CCK-8 assay.

A previous study reported that SCD1, a lipogenesis-related gene in mammals, plays a pivotal role in tumor inhibition. To explore whether H_2_ was involved in lipid metabolism in CRC, western blotting and IHC assays were performed to evaluate the status of SCD1 after H_2_ treatment in CRC cells. We found that SCD1 was markedly downregulated in H_2_ treatment CRC cells. The PI3K/AKT pathway controls various cellular activities, including proliferation, apoptosis, and autophagy. Therefore, we investigated the effect of H_2_ on the PI3K/AKT signaling pathway in CRC cells. We found that H_2_ obviously decreased the expression of phosphorylated AKT (pAKT) in three cell lines. Dramatically, the expression of PI3K was increased in RKO cell and decreased in SW480 and HCT116 cells (Figures 3(a) and 3(b)). To verify that H_2_ inhibits the proliferation of colorectal cancer through PI3K/AKT pathway, we treated RKO, SW480, and HCT116 cells with SC79, an AKT activator that increased pAKT expression in a dose-dependent manner. As expected, the inhibition of cell proliferation induced by H_2_ was reversed when all CRC cells were treated with SC79, as indicated in the CCK-8 assay (Figure 3(c)). Western blotting assays showed that the expressions of SCD1 and pAKT were significantly upregulated in a dose-dependent manner with SC79 treatment (Figure 3(c)). However, PI3K and p-PI3K showed different tendencies in these cells (Figure 3(b)). Together, these findings indicate that H_2_ inhibits tumor growth both *in vitro* and *in vivo* by targeting the pAKT/SCD1 pathway.

### 3.4. SCD1 Status and Associations with Clinicopathological Characteristics and Prognostic Value in CRC Patients

**Figure 4:**
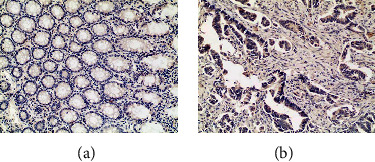
The expression of SCD1 in paraffin-embedded CRC and normal epithelium tissues. SCD1 expression levels in malignant cells were significantly higher than those in normal epithelial tissues (100x).

**Figure 5:**
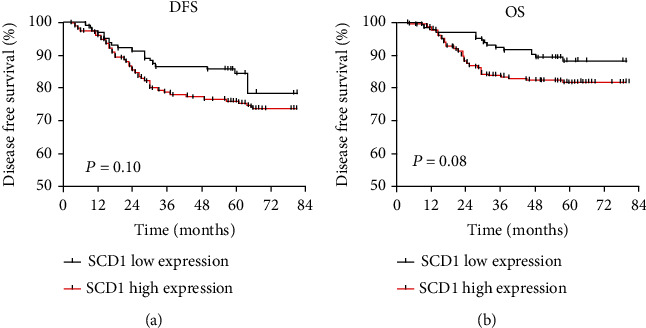
The prognostic value of SCD1 expression in primary CRC. Survival curves for disease free survival (DFS) and overall survival (OS) in stages I–III colorectal cancer according to SCD1 status: (a) DFS according to SCD1; (b) OS according to SCD1.

IHC staining was performed to analyze the expression of SCD1 in paraffin-embedded CRC and normal epithelium tissues in 491 CRC patients. As shown in Figure 4(a), SCD1 expression levels in malignant tumors were significantly higher than those in normal epithelial tissues. High expression of SCD1 was observed in 70.3% (345/491) of tumor tissues, but only 29.7% (146/491) of the normal epithelial tissues exhibited high SCD1 expression (Table 1). Correlations between SCD1 status and clinicopathological characteristics, including age, sex, tumor location, tumor size, histological characteristics, TNM stage, and family history, were analyzed (Table 1). Elevated SCD1 expression was significantly correlated with more advanced TNM stage (III vs I + II: 75.9% vs. 66.3%, *p* = 0.02), lymph node metastasis (with vs. without: 75.9% vs. 66.3%, *p* = 0.02), and no family history of CRC (78.7% vs. 62.1%, *p* = 0.047). Although tumor with higher expressed SCD1 was present more often in patients with mucinous tumors (with vs. without), there was no significant difference in this study (86.9% vs. 71.7%, *p* > 0.05). No significant differences between SCD1 expression and other clinicopathological characteristics were found in the present study. In addition, univariable analysis with Kaplan–Meier survival curves indicated that the expression of SCD1 was not related to DFS and OS (Figure 5).

## 4. Discussion

In recent years, the role of the tumor microenvironment in the occurrence and development of cancer has been widely recognized. Tumor cells prefer a prooxidative microenvironment, as antioxidants prevent tumors from achieving their most ideal redox level; these antioxidants decrease telomerase activation, thereby inhibiting tumor cell viability [24]. The mechanism by which molecular hydrogen controls disease involves its antioxidant and anti-inflammatory properties to reduce oxidative stress [25]. Therefore, the antitumor effect of hydrogen may be related to the improvement of the tumor microenvironment, and the inhibitory activity of molecular hydrogen on cancer has been reported in several types of tumors, including lung cancer [10], endometrial cancer [11], glioblastoma [12], and colon cancer [7]. Moreover, both low concentrations and high concentrations of hydrogen presented significant antitumor effects [11, 12]. In the present study, we found that a low concentration of hydrogen (30%) could kill SW480 cells, but only a high concentration of hydrogen (more than 50%) could inhibit RKO and HCT116 cell proliferation. SW480 and RKO cell lines are from different types of colon cancer: SW480 cells are derived from rectal cancer, and RKO cells are derived from colon adenocarcinoma. This indicates that different types of cells showed different responses to hydrogen based on the dose. Therefore, the appropriate concentration of hydrogen in cancer treatment needs to be verified by additional and more rigorous clinical studies. In addition, we observed that hydrogen did not promote apoptosis in CRC cells. Further investigation is still needed to ascertain the molecular mechanisms involved in the hydrogen-mediated control of proliferation in CRC cells.

With the development of related studies in hydrogen biomedicine, the traditional anti-inflammatory and antioxidant mechanisms are not enough to explain the inhibitory effects of hydrogen on cancer. Recently, Liu et al. [12] demonstrated that hydrogen can act on biological enzymes and promote acetylcholinesterase activity, thus reducing the production of toxic free radicals and inducing glioma cells to dedifferentiate into glial stem cells. Interestingly, the physical effect of hydrogen on CRC tumor stem cell differentiation remains largely unknown.

In mammalian cells, SCD1 is responsible for de novo synthesis of FAs, which are vital constituents in cellular processes, such as components of biological membranes and sources of energy and cell lipids (i.e., phospholipids, diacylglycerols, triacylglycerols, and cholesteryl esters). SCD1 expression is significantly elevated in various human cancer cells, including liver cancer [18], breast cancer [19], and colon cancer [26]. Moreover, the increased expression of SCD1 is positively correlated with cancer aggressiveness and poor patient prognosis [18, 19]. SCD1 has been identified as a novel key player in tumorigenesis and a potential target for anticancer therapy. The proposed underlying mechanisms of SCD1 in cancer involve multiple aspects: (a) inhibiting cell survival and proliferation by regulating lipid metabolism; (b) influencing the physiologic processes of cell cycle progression, apoptosis, and cell contact inhibition; and (c) promoting cancer stem cell (CSC) transformation [20]. A study conducted by Scaglia and Igal [27] demonstrated that knockdown of SCD1 in human lung cancer cells can decrease the rate of cell proliferation and induce apoptosis by decreasing MUFA and phospholipid synthesis. A recent analysis revealed that the SCD1-dependent regulation of FA, TAG, cholesterol, and PL synthesis was dependent on SREBP activation [28]. Yu et al. [26] found that SCD1 could induce CSC-specific apoptosis in colon cancer by targeting suppressed Notch and Wnt signaling pathways. These observations provided strong evidence that SCD1 is a crucial driver of neoplastic progression, and the application of SCD1 inhibitors may be an effective anticancer strategy. Additionally, a previous study provided evidence for the involvement of SCD1 in AKT phosphorylation. Scaglia and Igal [29] demonstrated that knockdown of SCD1 impaired lung cancer cells via inhibition of AKT phosphorylation. Holder et al. [30] showed that the expression of SCD1 is upregulated by PI3K/AKT signaling in breast cancer. In the present study, we provided the first evidence that hydrogen treatment could exert an antitumor effect against colorectal cancer cells by downregulating SCD1. Moreover, downregulated SCD1 was induced by decreased pAKT.

AKT, also known as protein kinase B (PKB), is a 57 kDa serine/threonine kinase and a critical mediator of growth factor-induced cell survival [31]. AKT can be phosphorylated, and pAKT levels are elevated in most malignant tumors. AKT activation plays an integral role in de novo lipid biosynthesis, fatty acid oxidation, and VLDL assembly and secretion in proliferating cells. Activation of AKT by phosphorylation can also suppress apoptosis and promote tumor proliferation. Therefore, pAKT may be a therapeutic target for cancer [32]. However, a previous study reported that a high concentration of H_2_ could improve heart ischemia/reperfusion injury in a mouse model by increasing the protein expression of p-AKT1 [33]. In our study, we found that hydrogen did not change the expression of total AKT but significantly decreased pAKT levels, indicating that hydrogen might suppress colorectal cancer cell proliferation by inducing pAKT (Ser473). To confirm our speculation, we exposed CRC cells to SC79 during hydrogen treatment. The results showed that the inhibition of cell proliferation induced by H_2_ was reversed by treatment with SC79 in all three cell lines. This result demonstrated that hydrogen inhibited colorectal cancer cell survival by reducing pAKT. We hypothesized that the mechanism of hydrogen-reduced AKT phosphorylation might be as follows: first, the intervention of hydrogen is likely to change the state of the hydrogen-bonding network in the microenvironment by rearranging the electrons, thus promoting the efficiency of energy transfer of the phosphorylation domain to inactive the enzyme [34–36]; second, hydrogen “disturbs” the hydrogen-bonding networks formed by “intramolecular” and “intermolecular” H_2_O and H_2_O_2_ and changes the enzyme activity bridged between “H_2_O_2_-H_2_-H_2_O” [37, 38], thus affecting the phosphorylation domain of AKT; and third, hydrogen might reduce certain kinds of lipids that are involved in lipid metabolism, resulting in inhibition of AKT activity [39]. Furthermore, we speculated that hydrogen suppressed the expression of SCD1 by reducing pAKT. We examined the expression of SCD1 in CRC cells after treatment with SC79, and the levels of SCD1 were markedly increased after SC79 treatment. These findings indicated that hydrogen decreased cell survival in CRC cells by targeting the pAKT/SCD1 pathway, and SCD1 may be a specific molecular target responsible for hydrogen biomedicine.

In addition, as an upstream marker of AKT, the expression of PI3K increased in RKO cell and decreased in SW480 and HCT116 cells. So, the different cells might show different effects on hydrogen. Hydrogen reduced the expression of PI3K and p-AKT in SW480 and HCT116 cells and increased the expression of PI3K in RKO cell. Thus, hydrogen may inhibit the PI3K/p-Akt pathways to reduce the proliferation in colorectal cancer cells. As for the increased expression of PI3K, we speculated that RKO cell enhanced expression of PI3K to resist the damage caused by inhibition of p-AKT. These results suggest that reduced phosphorylation of AKT might induce the inhibition of downstream signaling molecules in CRC cells, and the upstream signaling marker alteration caused by the reduced p-AKT might differ according to the molecular features and multiple and complicated effects caused by the cross-talk of each CRC cell. To determine the clinical relevance of SCD1, we performed IHC analyses of 491 paired samples of primary CRC and adjacent normal tissues. SCD1 was markedly increased in CRC specimens compared with normal tissues. High SCD1 expression was observed in 70.3% of tumor tissues, but only 29.7% of the normal epithelial tissues exhibited high SCD1 expression. We found that high SCD1 expression was correlated with more advanced TNM stage, lymph node metastasis, and patients without a family history of CRC. However, the survival curves indicated that the expression of SCD1 was not related to DFS and OS. Consistently, some studies have demonstrated that SCD1 can promote cancer cell proliferation by maintaining a population of cancer stem cells [21, 26]. Based on these findings, SCD1 plays an important role in the development of malignant disease and may be a promising target for anticancer therapy. Collectively, treatment with H_2_ may be appropriate to prevent recurrence and metastasis in CRC patients with tumors expressing high levels of SCD1.

In the present study, we explored the effects of hydrogen on CRC and provided the first evidence that hydrogen inhibits CRC cell proliferation via the pAKT/SCD1 pathway. We demonstrated that inhalation of 67% hydrogen gas reduced the volume and weight of CRC tumors in a xenograft mouse model. Our study suggests that inhalation of hydrogen is effective in treating CRC. Tumor tissue sections in the hydrogen group presented much less pAKT and SCD1 staining by IHC, and SCD1 expression was higher in CRC than in adjacent normal tissues. However, there are some limitations to our study. First, current knowledge demonstrates that SCD1 inhibits CRC proliferation by promoting apoptosis, mitochondrial dysfunction, ceramide synthesis, and stem cell differentiation [26, 28, 40], but we did not test the mechanism downstream of SCD1 in hydrogen-treated CRC cells. Second, we did not use the patient tissue-derived tumor xenografts, which were considered to be more accurately mimic human tumors with the high similarity in the tumor growth environment, so the effect of hydrogen in the orthotopic colorectal cancer model needs be further studied. Last, we did not determine the in vitro H_2_ concentration of the medium equivalent to the H_2_ concentration of the inhalation treatment used *in vivo*, so the H_2_ concentrations *in vitro* and *in vivo* may not have been equivalent.

## 5. Conclusion

The present study demonstrated that a high concentration of hydrogen exerts an inhibitory effect on CRC by inhibiting the pAKT/SCD1 pathway. SCD1 expression levels in malignant tissues were significantly higher than those in matched normal epithelial tissues in patients with CRC. Further studies are warranted for the clinical evaluation of hydrogen as an SCD1 inhibitor to target CRC.

## Figures and Tables

**Table 1:** SCD1 status and its association with clinicopathological characteristics in CRC patients.

SCD1 expression						
Characteristics	Number	High	(%)	Low	(%)	*p*
Gender						
Male	299	203	67.90%	96	32.10%	0.15
Female	192	142	73.90%	50	26.10%	
Age (years)						
≤50	64	41	64.10%	23	35.90%	0.24
>50	427	304	71.20%	123	28.80%	
Location						
Right side colon	112	76	67.80%	36	32.10%	0.53
Left side colon	93	58	62.40%	35	37.60%	
Rectum	286	211	73.80%	75	26.20%	
Mucin production						
With	46	40	86.90%	6	13.10%	0.07
Without	425	305	71.76%	120	28.20%	
Tumor differentiation						
Poor	106	68	64.20%	38	35.80%	0.12
Moderate/well	385	277	71.90%	108	28.10%	
Tumor diameter						
≤ 5 cm	269	190	70.60%	79	29.40%	0.85
>5 cm	222	155	69.80%	67	30.20%	
Tumor stage						
I + II	288	191	66.30%	97	33.70%	0.02
III	203	154	75.90%	49	24.10%	
Bowel wall invasion (T)						
T1 + T2	91	62	68.10%	29	31.90%	0.62
T3 + T4	400	283	70.80%	117	29.20%	
Lymph node metastasis (*N*)						
Without	288	191	66.30%	97	33.70%	0.02
With	203	154	75.90%	49	24.10%	
Lymphovascular invasion						
No	335	244	72.80%	91	27.20%	0.07
Yes	156	101	64.70%	55	35.30%	
Alcohol intake						
Never	387	275	71.10%	112	28.90%	0.46
Ever	104	70	67.30%	34	32.70%	
Smoking						
Ever	137	87	63.50%	50	36.50%	0.043
Never	354	258	72.90%	96	27.10%	
Cancer family history						
Yes	93	68	73.10%	25	26.90%	0.25
No	119	95	79.80%	24	20.20%	
Unknown	279					
Colorectal family history						
Yes	29	18	62.10%	11	37.90%	0.047
No	183	144	78.70%	39	21.30%	
Unknown	279					
MSI status						
MSI	68	46	67.60%	22	32.40%	0.61
MSS	423	299	70.70%	124	29.30%	
KRAS status						
Mutation	212	147	69.30%	65	30.70%	0.7
Wild type	279	198	70.90%	81	29.10%	

## Data Availability

The data used to support the findings of this study are available from the corresponding author upon request.

## Supplementary Materials

Supplementary Materials H_2_ did not promote apoptosis for CRC cells.
