# Supplementary figures and images for: Molecular Hydrogen Inhibits Colorectal Cancer Growth via the AKT/SCD1 Signaling Pathway
Source: Biomed Res Int. 2022 Apr 26;2022:8024452. doi: 10.1155/2022/8024452 (PMC9071919; doi:10.1155/2022/8024452)

**A****RKO****CTRL**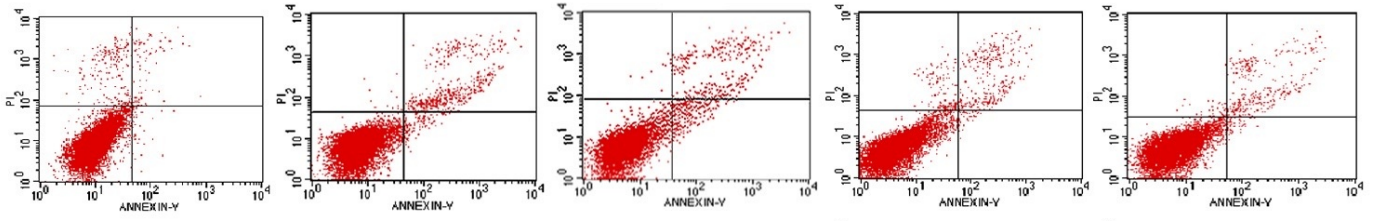**50% $H_2$** 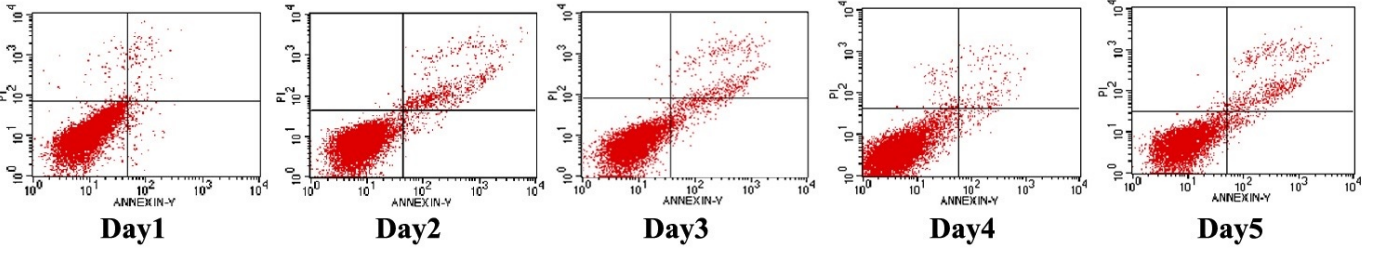**B****SW480****CTRL**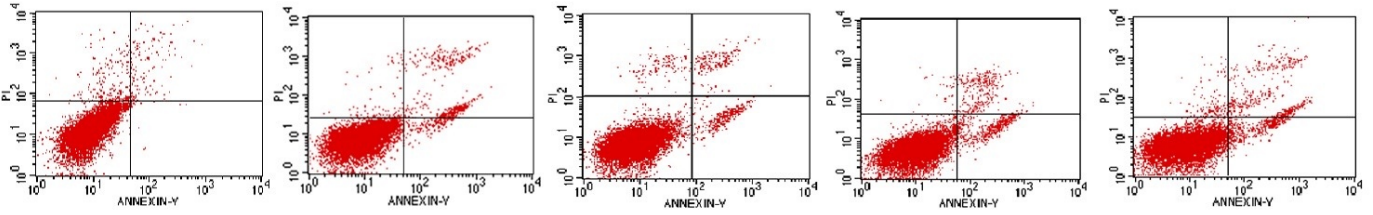**50% $H_2$** 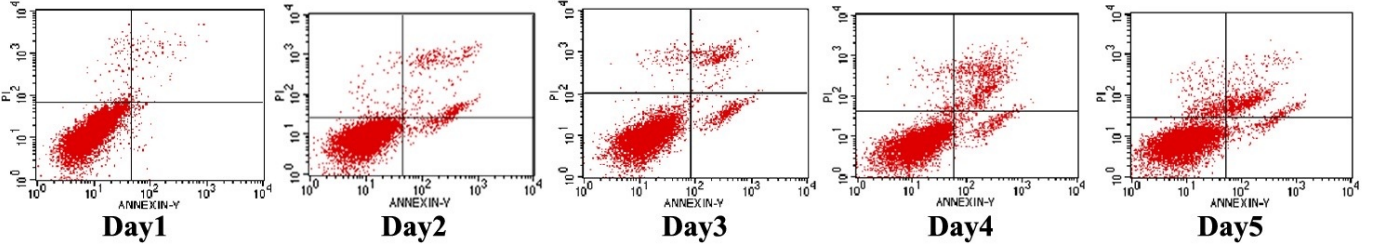

Supplement: Supplementary Materials — H2 did not promote apoptosis for CRC cells. [file 8024452.f1.pdf]
